# Idiopathic intracranial hypertension: Evaluation of births and fertility through the Hospital Episode Statistics dataset

**DOI:** 10.1111/1471-0528.17241

**Published:** 2022-06-21

**Authors:** Mark Thaller, Jemma Mytton, Benjamin R. Wakerley, Susan P. Mollan, Alexandra J. Sinclair

**Affiliations:** ^1^ Metabolic Neurology Institute of Metabolism and Systems Research University of Birmingham Birmingham UK; ^2^ Department of Neurology University Hospitals Birmingham NHS Foundation Trust Birmingham UK; ^3^ Informatics University Hospitals Birmingham NHS Foundation Trust Birmingham UK; ^4^ Birmingham Neuro‐Ophthalmology University Hospitals Birmingham NHS Foundation Trust Birmingham UK; ^5^ Centre for Endocrinology, Diabetes and Metabolism, Birmingham Health Partners Birmingham UK

**Keywords:** birth, caesarean section, epidemiology, fertility, Hospital Episode Statistics, idiopathic intracranial hypertension, papilloedema, polycystic ovarian syndrome, pregnancy, pseudotumor cerebri

## Abstract

**Objective:**

Idiopathic intracranial hypertension (IIH) predominantly affects women of reproductive age with obesity, and these women have a distinct profile of hyperandrogenism and insulin resistance. Polycystic ovary syndrome (PCOS) has an established adverse fertility phenotype that typically affects obese women. As IIH may impact reproductive health, we sought to evaluate fertility, gestational complications and pregnancy outcome in IIH.

**Design:**

Prospective cohort study from English Hospital Episode Statistics dataset.

**Setting:**

English hospitals, UK.

**Population:**

Women aged 18–45 years seen in English hospitals between 1 April 2002 and 31 March 2019. Patients were required to have an IIH diagnosis and were compared with those with PCOS and general population female controls.

**Main outcome measures:**

Pregnancies resulting in live births, complications of gestational diabetes and pre‐eclampsia, and method of delivery.

**Results:**

Data was collected from 17 587 IIH, 199633 PCOS and 10 947 012 women in the general population. The live birth rate, adjusted for age, was significantly lower among women with IIH (54.1%) than PCOS (67.9%), *p* < 0.0001 and the general population (57.7%), *p* < 0.0001. Pre‐eclampsia and gestational diabetes risks were higher following a diagnosis of IIH (5.3‐fold and 2.7‐fold, respectively, *p* < 0.0001) compared with the general population controls. Following a diagnosis of IIH, elective caesarean section rates were more than twice that of general population (odds ratio [OR] 2.4) and prior to a diagnosis of IIH (OR 2.2).

**Conclusions:**

These data indicate there are lower age‐adjusted total pregnancy rates, increased risk of pre‐eclampsia and gestational diabetes, and a doubling of elective caesarean section rates in those with a diagnosis of IIH.

## Abstract 2

This article includes Author Insights, a video abstract available at: https://vimeo.com/bjogabstracts/authorinsights17241

## 1 INTRODUCTION

Idiopathic intracranial hypertension (IIH) is a disorder of raised intracranial pressure typically manifesting in reproductive‐aged women with obesity. The incidence in women has increased by over 350% in the last decade to 9.3 per 100 000.^1^, ^2^, ^3^, ^4^ Typical manifestations of the disease are debilitating headaches and visual loss,^5^, ^6^ with many having cognitive dysfunction^7^ and obstructive sleep apnoea.^8^ More recently, evidence has pointed towards a condition of metabolic dysregulation.^9^ IIH patients have preferentially distributed truncal adiposity^10^ as well as increased risk of cardiovascular disease^3^ and insulin resistance in excess of that driven by obesity.^11^ IIH adipocyte function demonstrates a distinct transcriptional profile with adipocytes programmed for lipogenesis and weight gain.^11^

IIH patients have a unique hormone signature characterised by androgen excess with increased serum testosterone, and increased CSF testosterone and androstenedione, which has been found to be distinct from that observed in polycystic ovarian syndrome (PCOS) and simple obesity.^12^ IIH is phenotypically similar to PCOS, with both conditions typically occurring in women of reproductive age with obesity. A community‐based PCOS study found a self‐reported association of a 15‐fold increase in infertility.^13^ The impact of IIH on reproductive health is not known and is an area declared as a high priority by patients.^14^ We hypothesised that fertility may be reduced in IIH, there may be an increase in the metabolic complications of gestational diabetes and pre‐eclampsia, and that there may be an increased surgical delivery rate. The aims of this study were to evaluate the impact of a diagnosis of IIH on women's fertility rates, pregnancy complications (gestational diabetes mellitus and pre‐eclampsia) and method of delivery using the English National Health Service (NHS) Hospital Episode Statistics (HES) dataset.

## 2 METHODS

### 2.1 Study design

Data were obtained from the English National Health Service (NHS) Hospital Episode Statistics (HES) dataset, a registered administrative dataset. All women aged between 18 and 45 years, admitted to all hospitals (both private and public) in England between 1 April 2002 and 31 March 2019 were selected. Clinical episodes are defined as admissions to ambulatory care (e.g. for lumbar punctures), emergency room visits and inpatient care. Each clinical episode taking place in National Health Service (NHS) hospitals or NHS commissioned activity in the independent sector was recorded. Each record was anonymised and comprised specific demographic details of the admitted patient including age group, gender, ethnicity and geographical information such as the location of treatment and domicile. Coding was either ICD‐10 (International Classification of Diseases, 10th revision)^15^ or OPCS‐4 (Office of Population, Censuses and Surveys Classification of Interventions and Procedures, 4th revision) codes.^16^ Body mass index (BMI) is not recorded within HES data.

University Hospitals Birmingham NHS Foundation Trust possesses a Data Re‐Use Agreement for the interrogation of the HES. The research involved non‐identifiable information, previously collected during patient care and available for public use. Where there are <5 people in any category, the results were not made available to ensure anonymisation was upheld. University Hospitals Birmingham National Health Service Foundation Trust approved this study (Registered Code, Clinical Audit Registration and Management System: CARMS‐17157).

A diagnosis of IIH in the UK is made by the hospital specialist. Typically, they should follow the consensus guidelines for this diagnosis, which include the presence of papilloedema; normal neurological examination except for cranial nerve abnormalities; normal neuroimaging (except for the accepted signs of raised intracranial pressure) including venography; a raised lumbar puncture opening pressure (≥25 cm cerebrospinal fluid [CSF] in adults); and with normal CSF constituents.^6^ This diagnosis is coded by the administrative staff and transmitted to the HES data set on a yearly basis. While the inclusion criteria for diagnosis are detailed to 31 March 2019, pregnancy outcome data was extracted up to 31 March 2020 so that all participants had at least a 1‐year follow‐up.

**FIGURE 1:**
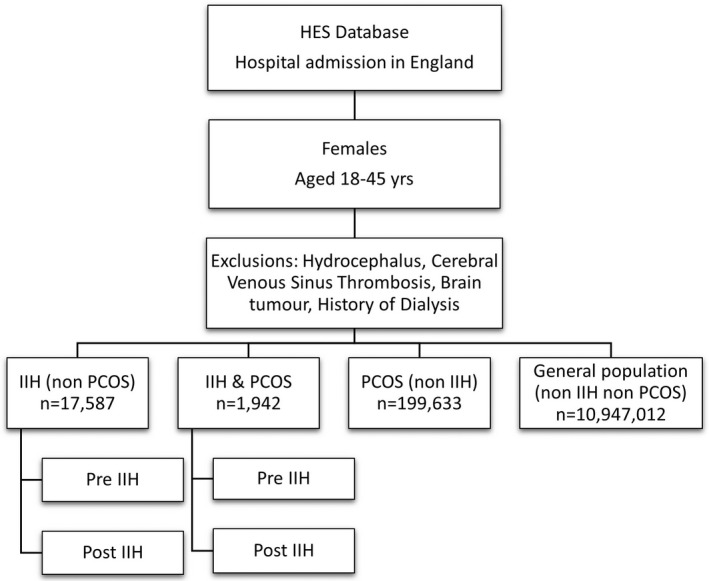
CONSORT diagram for patient groups included in this study.

Three further groups were extracted for comparison purposes with the same age range and time frame: women diagnosed with PCOS and no IIH (PCOS), women with both IIH and PCOS diagnoses (IIH & PCOS) and women admitted for any other reason without IIH and without PCOS (general population) (Figure 1). Exclusion criteria were applied to help refine the data and ensure against miscoding of secondary causes of raised intracranial pressure such as brain tumours, hydrocephalus and cerebral venous sinus thrombosis. Due to the very high number of admitted patient care episodes and comorbidities, we excluded those with a history of dialysis, as these were likely to represent a secondary cause of raised intracranial pressure and the high admission rates would have potentially biased the results. Those who resided outside of England or whose residence was unknown were also excluded, as accurate longitudinal patient tracking was not always possible. Date of diagnosis for IIH (first coded) was used to enable comparison of data before (pre‐IIH) and after IIH diagnosis (post‐IIH). Patients were not directly involved in this study.

### 2.2 Data collection

All coded pregnancies, births and losses (miscarriages and stillbirths) were collected. Table S1 lists the ICD‐10 or OPCS‐4 codes included and excluded from this study. The data were analysed by age at pregnancy and number of pregnancies/births/losses per female. Fertility was evaluated using a surrogate measure of the number of women without any pregnancies during this study period, as well as the total fertility rate, which is age‐adjusted and based on the age‐specific fertility rate.

Gestational complications of gestational diabetes (GDM) and pre‐eclampsia were compared, with gestational hypertension without proteinuria (ICD10 O13) alone excluded from the latter. Delivery method (normal live delivery, assisted live delivery, emergency or elective caesarean section [CS]) and number of births were also retrieved. The data were divided by the occurrences pre‐ or post‐IIH diagnosis for both the IIH and IIH & PCOS groups. The codes used for inclusion, exclusion and outcomes are listed in Table S1.

### 2.3 Statistical analysis

The data were initially explored through descriptive analysis of variables using *t*‐tests for quantitative variables and McNemar's *binomial* tests for categorical variables to compare different groups. Poisson regression analysis was used to model the total number of pregnancies per patient with adjustments for age at first pregnancy and timing of IIH diagnosis in relation to their first pregnancy. Patients were included in this analysis if they had at least one pregnancy. Odds ratios (OR) were calculated using the Woolf logit method^17^ through logistic regression to enable comparison in complication and method of delivery outcomes between the IIH, PCOS and general population groups.

All statistical analyses were conducted using GraphPad Prism™ (version 9.1.0) with level of statistical significance set a *p* < 0.5.

## 3 RESULTS

### 3.1 Demographics

Pregnancy data were analysed for 17 587 patients diagnosed with IIH, 199 633 with PCOS, 1942 IIH&PCOS, and 10 947 012 in the general population. In the general population, the incidence of IIH per 100 000 women was 3.1, PCOS was 44.8, and IIH with comorbid PCOS was 0.36 (2018–2019). Mean age at pregnancy was similar post‐IIH diagnosis, PCOS and the general population (Table 1).

**TABLE 1:** Demographics, pregnancy complications and assisted delivery method. The complication and assisted delivery percentages are for those who had at least one pregnancy

		IIH		PCOS	IIH & PCOS		General population
		Pre‐IIH	Post‐IIH		Pre‐IIH	Post‐IIH	
Demographics	Females 18–45 years, *n*	17 587		199 633	1942		10 947 012
	Pregnancies, *n*	12 419	7261	277 067	1262	918	11 848 846
	Pregnancy losses, *n* (%)	1017 (8.2%)	548 (7.5%)	35 177 (12.7%)	160 (12.7%)	98 (10.7%)	962 828 (8.1%)
	Females with zero pregnancies, *n* (%)	10 607 (60.3%)	12 774 (72.6%)	59 860 (30.0%)	1221 (62.9%)	1319 (67.9%)	4 464 198 (40.8%)
	Age at pregnancy, mean (SD)	25.0 (5.1)	28.6 (5.0)	29.0 (5.5)	24.7 (4.8)	29.4 (4.9)	29.6 (5.8)
Complications	Gestational diabetes, *n* (%)	310 (4.4%)	591 (12.3%)	16 444 (11.8%)	59 (8.2%)	128 (20.6%)	322 596 (5.0%)
	Pre‐eclampsia, *n* (%)	290 (4.2%)	570 (11.8%)	6055 (4.3%)	41 (5.7%)	59 (9.5%)	161 546 (2.5%)
Assisted delivery	Assisted vaginal delivery, *n* (%)	1067 (9.4%)	528 (7.9%)	29 405 (12.2%)	122 (10.2%)	57 (6.9%)	1 302 447 (12.0%)
	Elective caesarean section, *n* (%)	1266 (11.1%)	1500 (22.3%)	30 275 (12.5%)	125 (11.3%)	224 (27.3%)	1 171 464 (10.8%)
	Emergency caesarean section, *n* (%)	1900 (16.7%)	1325 (19.7%)	44 231 (18.3%)	199 (18.1%)	199 (24.2%)	1 615 195 (14.8%)

### 3.2 Fertility and pregnancy

During the study, 19 680 pregnancies were reported in women who were diagnosed with IIH, compared with 277 067 in PCOS, 2180 in IIH + PCOS, and 11 848 846 in the general population. Pregnancy losses (miscarriage or stillbirth) were reported in 1565 IIH, 35 177 PCOS, 258 IIH + PCOS, and 962 828 in the general population.

**FIGURE 2:**
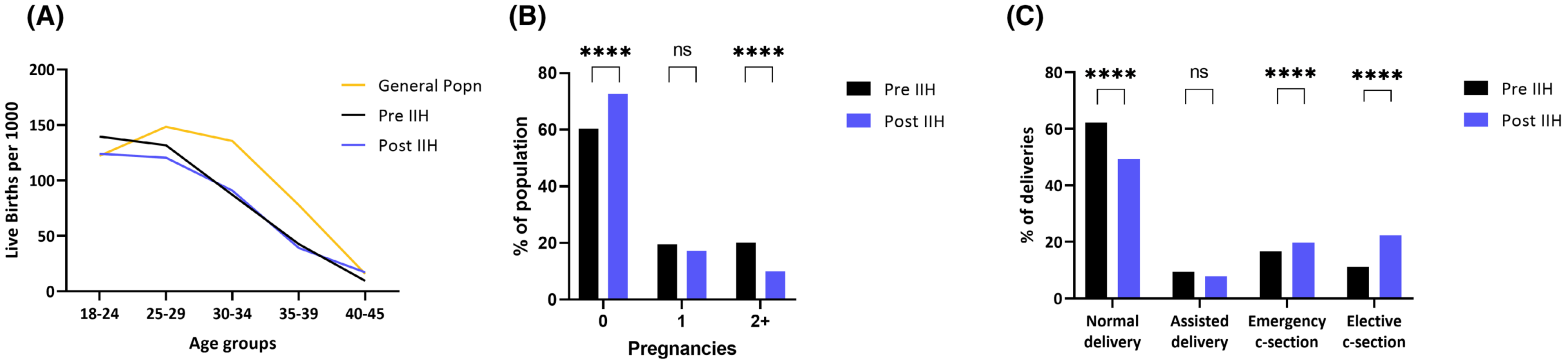
(A) Age‐specific fertility rate in IIH (pre‐ and post‐diagnosis) and general population in 2015. (B) Impact on pregnancy numbers following an IIH diagnosis. (C) Impact on delivery method following an IIH diagnosis. ****McNemar *p* < 0.0001.

In the childbearing ages between 25 and 39 years, the age‐specific fertility rate (Figure 2A) was lower in IIH, regardless of whether it was pre‐ or post‐IIH diagnosis, compared with the general population. After adjusting for age at first pregnancy confounder, through Poisson regression modelling, the IIH pregnancy rate was lower in IIH than in the general population (incidence rate ratio (IRR) 0.53 (0.52–0.54) (*p* < 0.001), indicating that this is not an age effect. The predicted total number of pregnancies in IIH was 0.95 (0.94–0.96) in contrast to 1.80 (1.80–1.80) for the general population.

Following an IIH diagnosis, the number of pregnancies decreased by 41.5% from 12 419 to 7261 (Table 1). Following diagnosis of IIH in patients with comorbid PCOS, pregnancies decreased by 27.2% from 1262 to 918. Following a diagnosis of IIH, women were less likely to have a further pregnancy compared with prior to an IIH diagnosis: IRR 0.83 (0.80–0.86), *p* < 0.001. There were fewer women with ≥2 pregnancies post‐IIH diagnosis (1775, 10.1%) than pre‐IIH diagnosis (3553, 20.2%) (Figure 2B) despite a similar mean age pre‐ and post‐IIH diagnosis (Table 1). Infertility, inferred by the absence of pregnancies, occurred in 60.3% of women pre‐IIH diagnosis and increased to 72.6% (*p* < 0.0001) post‐IIH diagnosis (Figure 2B, Table 1), in comparison with 40.8% in the general population.

### 3.3 Timing of IIH diagnosis and pregnancy

We explored the timing of IIH diagnosis and pregnancy. Where there was a pre‐IIH diagnosis pregnancy, the last pregnancy occurred more than 3 years previously in 62.5%. Where IIH was diagnosed during pregnancy, this was predominantly in the second and third trimester (1035 [5.9%] of IIH patients overall) with a reduced diagnostic proportion (219 [1.2%]) during the immediate 6 months postpartum.

### 3.4 Complications

**FIGURE 3:**
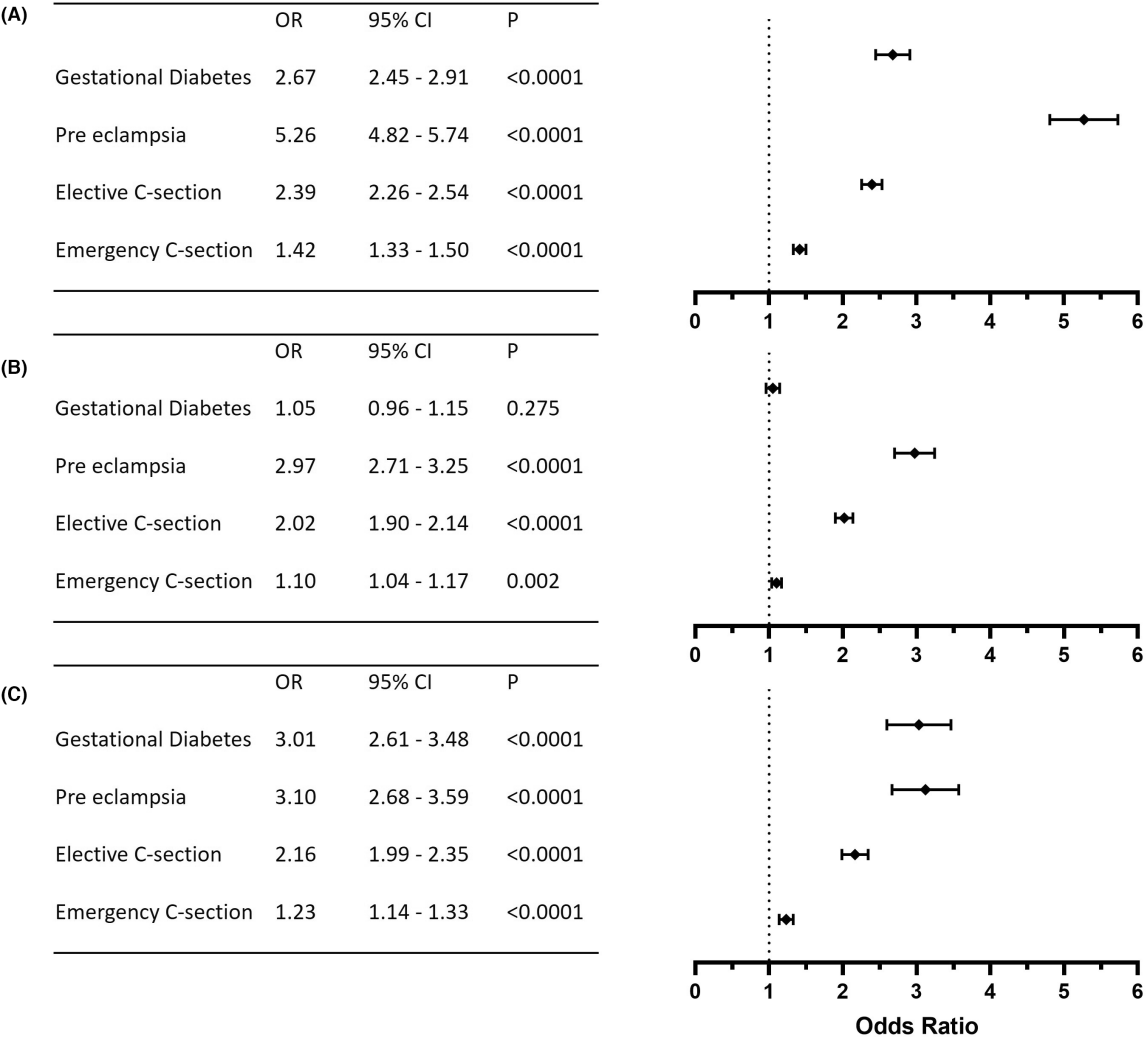
Odds ratio (OR), 95% confidence interval (CI), *p*‐values post‐IIH diagnosis compared with (A) general population, (B) PCOS and (C) pre‐IIH.

Gestational diabetes was higher in IIH (12.3%) and PCOS (11.8%) patients compared with the general population (5.0%) (Table 1). In women prior to a diagnosis of IIH, gestational diabetes was lower (4.4%). The risk of developing gestational diabetes was higher following an IIH diagnosis (OR 3.01 [2.61–3.48], *p* < 0.0001) and when compared with the general population (OR 2.67 [2.45–2.91], *p* < 0.0001) (Figure 3), but there was no difference compared with patients with PCOS (*p* = 0.2751).

Pre‐eclampsia was higher in IIH patients (11.8%), compared with the general population (2.5%), PCOS patients (4.3%) and prior to a diagnosis of IIH (4.2%). The probability of developing pre‐eclampsia in IIH was higher than in the general population (OR 5.26 [4.82–5.74], *p* < 0.0001), PCOS (OR 2.97 [2.71–3.25], *p* < 0.0001) or compared with those prior to a diagnosis of IIH (OR 3.10 [2.68–3.59], *p* < 0.0001) (Figure 3).

### 3.5 Delivery method

Rates of assisted vaginal delivery, elective and emergency caesarean section were evaluated (Figure 2C). We found that the occurrence of elective caesarean section doubled following IIH diagnosis (from 11.1% to 22.3%) (Figure 2C), with the probability being statistically significantly higher (OR 2.16, 95% confidence interval [CI] 1.99–2.35, *p* < 0.0001). Additionally, the probability of elective caesarean section was statistically significantly higher compared with the general population (OR 2.39, 95% CI 2.26–2.54, *p* < 0.0001) and PCOS groups (OR 2.01, 95% CI 1.90–2.14, *p* < 0.0001) (Figure 3). The highest probability of elective caesarean section was among those with both IIH and PCOS (OR 3.12, 95% CI 2.67–3.64, *p* < 0.0001). Emergency caesarean section was also consistently higher in patients with IIH (Figure 3).

## 4 DISCUSSION

### 4.1 Main findings

This large observational study found that patients with IIH had statistically significantly fewer pregnancies, more pregnancy‐related complications (gestational diabetes and pre‐eclampsia) and were more likely to have caesarean sections. Although fertility normally decreases with increased age, this study indicates that age‐specific fertility rates at the vast majority of time points were lower in IIH both pre‐ and post‐diagnosis compared with the general population.

### 4.2 Interpretation

This is the first study to report that people with IIH may have reduced fertility. The cause of this reduction is likely to be multifactorial. Obesity is known to affect fertility.^10^, ^18^, ^19^ It increases the risk of menstrual irregularities, typically as a result of anovulation.^19^ This is through decreased luteinising hormone pulse amplitude,^20^ which can lead to abnormal oocyte recruitment and quality.^21^ Circulating adipokines secreted by adipose tissue, i.e. leptin and TNF‐α, have been shown to vary over the menstrual cycle^22^, ^23^, ^24^ and influence multiple levels of the hypothalamic–pituitary–gonadal axis^18^, ^19^, ^20^ prolonged hyperleptinaemic states may therefore impact fertility.^24^ Hyperleptinaemia in excess of that driven by obesity has been shown in IIH^11^ and may influence fertility in IIH. These findings are unlikely to be driven by obesity alone, as we have compared with them a hospital‐based PCOS population that also is typically associated with obesity.

Metabolic syndrome is associated with older age at pregnancy and infertility, independent of obesity.^25^ IIH is a condition characterised by metabolic dysfunction with truncal adiposity as well as insulin resistance and twice the risk of cardiovascular disease in excess of that driven by obesity.^3^, ^11^

The hyperandrogenism in IIH^12^ may also contribute to reproductive dysfunction, as it is hypothesised to in PCOS.^13^, ^26^ In PCOS, reproductive function improves with dedicated treatment; further investigation for people with IIH may be benefical.^27^, ^28^ Treating metabolic perturbations with glucagon‐like peptide‐1 (GLP‐1) receptor agonists has been shown to improve menstrual regularity and increase fertility rates in women with PCOS and obesity.^29^ GLP‐1 receptor agonists are emerging as a potential treatment option for IIH, with preclinical data demonstrating their ability to reduce ICP and clinical efficacy data emerging.^30^, ^31^

A change in behaviour in people with a new diagnosis of IIH may contribute to the observed reduction in overall pregnancies and multiple parity. Behaviour change may be impacted by interactions with and advice from health professionals. There may be concerns that pregnancy could drive weight gain, a known risk factor for exacerbation of IIH.^32^, ^33^ Targeting disease remission prior to conception is recommended and hence people with IIH may wish to delay their pregnancy until this has been achieved.^33^ Indeed, medicines used to lower intracranial pressure have potential teratogenic risks (acetazolamide or topiramate), which may alter family planning decisions following a diagnosis.^6^, ^33^

We observed greater pregnancy‐related complications in patients with IIH. This in part may be explained by pre‐pregnancy obesity and excessive weight gain in early pregnancy, which are known to increase the risk of gestational diabetes and pre‐eclampsia.^34^ The increase in gestational diabetes and pre‐eclampsia in IIH is likely to be driven by metabolic dysregulation, although the precise mechanism has yet to be established. Metabolic syndrome is an established risk factor for gestational diabetes and pre‐eclampsia.^35^ Compared with PCOS, the gestational diabetic risk was not statistically significantly different and this likely reflects similar metabolic profiles, including a hyperandrogenic component, independent of obesity.^36^ Pre‐eclampsia in IIH, however, warrants further investigation to establish the pathophysiological process, as this risk appears to be greater in people with IIH than in those with PCOS. The increased risk of complications in IIH are of clinical significance, as they highlight the need for increased surveillance during pregnancy.^6^ Pregnancy losses in IIH were comparable to the general population, unlike in PCOS, where they are increased (Table 1); this reflects previous PCOS literature with a reported OR of 1.71 (1.19–2.45).^36^ This should reassure IIH patients that there is no increased risk.

Maternal obesity is known to increase operative delivery rates^37^ and excessive gestational weight gain increases the caesarean probability by 30%.^38^ Elective and emergency caesarean sections were statistically significantly more likely to occur in IIH as suggested by previous literature.^2^ The doubling of the elective rates is of clinical significance, as these are not due to perinatal complications but are rather a planned decision prior to labour. It remains unclear whether patient and doctor concerns about normal vaginal delivery in IIH play an important role. However, raised BMI, as a cause of this increased caesarean section rate, is unlikely to be the dominant driver, given higher rates than the phenotypically similar PCOS (Figure 3B). The literature reports that caesarean section rates are noted to be higher in PCOS than in obesity‐matched cohort studies (OR 1.55 [1.13–2.10])^36^ or PCOS with gestational diabetes (OR 1.72 [1.26–2.37]).^36^, ^39^, ^40^, ^41^ This indicates that factors beyond maternal obesity are likely to contribute to operative delivery rates in these hyperandrogenic disorders. The second stage of labour only transiently elevates intracranial pressure and is unlikely to impact on optic nerve function, except in the rare case of a pregnant person with fulminant IIH. There is therefore little evidence for an elective caesarean section purely on the basis of an IIH diagnosis alone.^6^, ^33^, ^42^

### 4.3 Strengths and limitations

Idiopathic intracranial hypertension is a rare disease but the incidence is increasing. The HES data source enables large sample analysis on a national basis, which in turn assists recognition of trends potentially not identifiable from a single hospital data source. The 11 million female general population controls enable comparisons with an unaffected cohort. The additional PCOS cohort facilitates comparisons with a population predisposed to obesity with known fertility and gestational problems. We adjusted for age, as this is a known confounder for fertility and gestational complications.

There are limitations to HES data, as it is reliant on clinical record keeping and coding.^43^ It may be sensitive to inconsistencies,^44^, ^45^ but method of delivery has been reported to be coded consistently.^46^ Deliveries and births are linked through HES and 98% remain linked^44^ with missing data reducing over time. Previous historical data for delivery method stated 15% missing data;^45^ however, in a separate study the missing delivery method was only 0.8%.^46^ We cannot ensure that every person fulfilled the diagnostic criteria for IIH; however, the diagnosis was made in a hospital setting. IIH HES data^1^, ^2^ has comparable rates of incidence and prevalence to the primary care data from evaluation of The Health Improvement Network (THIN) database.^3^

When interrogating HES data, it is important to be cognisant that for some diseases there may be inherent bias due to a lack of clear clinical criteria that is used by clinicians for a particular diagnosis (for example, the difference between primary pre‐eclampsia and superimposed pre‐eclampsia). The HES dataset is hospital‐based, therefore community diagnoses that do not result in hospital review or management would be underestimated, as in PCOS. This may account for the differences seen in PCOS fertility here (Table 1) compared with self‐reported PCOS rates of infertility in the literature.^13^ This would also be the case for early miscarriages that may not need hospital or day case admissions. In addition there may be unseen coding bias, for example an emergency and semi‐elective caesarean delivery does not have an international definition and has the potential to be prone to variability in coding between hospitals.

Numerical data, e.g. BMI values, are not part of this data source and so BMI could not directly be adjusted for; however, comparison was made to a PCOS population who share an obesity phenotype with IIH, therefore controlling in part for an obesity effect. Similarly those patients who do not attend hospital would not be accounted for. Infertility defined by the age‐specific fertility rate is not perfect because it does not account for an individual's relationship status or choice not to have children. Sensitive data, such as Reproductive Medicine codes for In Vitro Fertilisation, are not recorded within HES, so we are unable to establish the role of fertility treatments played in the fertility rates shown. Ectopic pregnancies were not specifically analysed in this study.

Analysing large datasets can be prone to type 1 statistical error. However, here we have provided descriptive outcomes rather than analysing efficacy data. The clinical context of the interpretation of the results remains important. HES offers the opportunity to find signals in rare diseases such as IIH that require further research in prospective studies to validate the findings.

## 5 CONCLUSION

Women with IIH had lower age‐adjusted total pregnancy rates than those with PCOS and the general population. Pregnancy complication risks, including pre‐eclampsia and gestational diabetes, were much higher in IIH. Following a diagnosis of IIH, elective caesarean section rates were more than doubled. A number of factors may contribute to these findings, including patient choice and systemic metabolic dysfunction. Specialist input to support reproductive health in IIH may improve outcomes.

## ACKNOWLEDGEMENTS

We would like to thank the patient charity IIHUK for their wider support of our research, as well as the IIH patients who support our ongoing research.

## AUTHOR CONTRIBUTIONS

MT, JM: acquisition of data; interpretation of data; drafting/revision of the manuscript for content. BRW: interpretation of data; drafting/revision of the manuscript for content. SPM: drafting/revision of the manuscript for content, including medical writing for content; and interpretation of data; AJS: study concept and design; interpretation of data; drafting/revision of the manuscript for content.

## Funding information

AJS is funded by a Sir Jules Thorn Award for Biomedical Science. The funding organisation had no role in the design or conduct of this research.

## CONFLICT OF INTERESTS

Dr Thaller and Ms Mytton report no conflicts of interest.; Dr Wakerley reports consultancy fees (Invex therapeutics); Professor Mollan reports consultancy fees (Invex Therapeutics; Neurodiem; Velux Foundation); advisory board fees (Invex Therapeutics; Janssen; Santhera) and speaker fees (Heidelberg engineering; Chugai‐Roche Ltd; Allergan; Santen; Chiesi; Santhera), all outside the submitted work. Professor Sinclair reports personal fees from Invex Therapeutics during the conduct of the study as well as share options and shareholdings; grants and funding for other trials; and speaker fees (Novartis; Allergan; Teva UK). Completed disclosure of interest forms are available to view online as supporting information.

## ETHICAL APPROVAL

No ethics approval was required for this study. University Hospitals Birmingham National Health Service Foundation Trust approved this study (Registered Code, Clinical Audit Registration and Management System: CARMS‐17157).

## Supporting information


Table S1

ICMJE

ICMJE

ICMJE

ICMJE

ICMJE

## Data Availability

Alex Sinclair takes full responsibility for the data, the analyses and interpretation, and the conduct of the research. Alex Sinclair has full access to all of the data; and has the right to publish any and all data separate. The data that support the findings of this study are available from the corresponding author upon reasonable request.
