# Supplementary material for: Idiopathic intracranial hypertension: Evaluation of births and fertility through the Hospital Episode Statistics dataset
Source: BJOG. 2022 Jun 21;129(12):2019–27. doi: 10.1111/1471-0528.17241 (PMC9796176; doi:10.1111/1471-0528.17241)
Supplement: Supplementary file 1 — Table S1 [file BJO-129-2019-s005.docx]

**Supplementary Table 1**

**Pregnancy in IIH Coding**

**IIH diagnosis**

- ICD10 G932

**Exclusions**

- Hydrocephalus (ICD10 G91)
- Cerebral Venous Sinus Thrombosis (ICD10 G08)
- Brain Tumour (ICD10 C70, C71)
- History of Dialysis (OPCS X401, X402, X403, X406) (ICD10 Z992, Z49)

**PCOS diagnosis**

- ICD10 E282

**Pregnancy coding**

- Normal Spontaneous Delivery (ICD10 O80 or OPCS R249)
- Instrumental Delivery (ICD10 O81 or OPCS R21, R22)
- Elective C Section (ICD10 O820 or OPCS R17)
- Emergency C Section (ICD10 O821 or OPCS R18)
- Multiple Deliveries (ICD10 O84)
- Outcome of Delivery (ICD10 Z37)
- Maternal Care for Intrauterine Death (ICD10 O364)
- Missed Abortion (ICD10 O021)
- Spontaneous Abortion (ICD10 O03)

**Gestational diabetes**

- ICD10 O244
- ICD10 O249

**Pre-eclampsia**

- ICD10 O13 (gestational hypertension without proteinuria)
  - Excluded from pre-eclampsia analysis
- ICD10 O140
- ICD10 O141
- ICD10 O149
